# 

**Published:** 1921-12

**Authors:** 


					CONTENTS. Page
Some Phases of Quackery in Relation to Diseases of the Eye.
n r*  IJT ^r.TT?TT, M R R A fintoh
By Cyril H. Walker, M.B., B.A. Cantab.
129
The Lone; Fox Lecture : The Repair of Bone Injuries.
? T*T T*t  /-?  -A * O TD O
By
Ernest W. Hky Groves, M.S., F.R.C.S.
142
The Role of Dilute Acids in Infection.
By I. Walker Hall and
A. D. Fraser ...
x58
Reviews of Books
163
Editorial Notes
i<r7"0l\ Cor
Obituary:
George Frederick Rossiter   I FEB 27
922
Local Medical Notes
The Library of the Bristol Medico-Chirurgical S/^39091I%\

				

